# An Automatic Head Surface Temperature Extraction Method for Top-View Thermal Image with Individual Broiler

**DOI:** 10.3390/s19235286

**Published:** 2019-11-30

**Authors:** Xingguo Xiong, Mingzhou Lu, Weizhong Yang, Guanghui Duan, Qingyan Yuan, Mingxia Shen, Tomas Norton, Daniel Berckmans

**Affiliations:** 1 College of Engineering/Jiangsu Province Engineering Lab for Modern Facility Agriculture Technology & Equipment, Nanjing Agricultural University, Nanjing 210031, China; 2 Jiangsu Lihua Animal Husbandry CO., LTD., Changzhou 213168, China; 3 M3-BIORES- Measure, Model & Manage Bioresponses, KU Leuven, Kasteelpark Arenberg 30, B-3001 Leuven, Belgium; 4 BioRICS nv, Technologielaan 3, 3001 Leuven, Vlaams-Brabant, Belgium

**Keywords:** broiler surface temperature extraction, thermal image processing, head region locating, adaptive K-means, ellipse fitting

## Abstract

Surface temperature variation in a broiler’s head can be used as an indicator of its health status. Surface temperatures in the existing thermograph based animal health assessment studies were mostly obtained manually. 2185 thermal images, each of which had an individual broiler, were captured from 20 broilers. Where 15 broilers served as the experimental group, they were injected with 0.1mL of pasteurella inoculum. The rest, 5 broilers, served as the control group. An algorithm was developed to extract head surface temperature automatically from the top-view broiler thermal image. Adaptive K-means clustering and ellipse fitting were applied to locate the broiler’s head region. The maximum temperature inside the head region was extracted as the head surface temperature. The developed algorithm was tested in Matlab^®^ (R2016a) and the testing results indicated that the head region in 92.77% of the broiler thermal images could be located correctly. The maximum error of the extracted head surface temperatures was not greater than 0.1 °C. Different trend features were observed in the smoothed head surface temperature time series of the broilers in experimental and control groups. Head surface temperature extracted by the presented algorithm lays a foundation for the development of an automatic system for febrile broiler identification.

## 1. Introduction

Body temperature is one of the most important indicators of a broiler’s health status [1]. When a broiler is infected with bacteria or a virus, the autoimmune system will take effect, resulting in an obvious rise of core body temperature [2]. Therefore, sudden changes in core body temperature can be utilized to identify a sick broiler. Traditional body temperature measurement is achieved by inserting a mercury thermometer into a broiler’s rectum [3]. It is laborious and time consuming. At the same time, it could increase the probability of disease spreading between farmers and broilers.

With the development of sensor technology, animal body temperature monitoring based on implantable temperature sensors has been evaluated in the rumen [4,5], vagina [6], and subcutis [7]. However, it is difficult to apply implantable sensors to collect body temperature for animals of small size, such as broilers. A wearable temperature sensor, positioned under a broiler’s wing, was utilized by Li, et al. [8] in 2013 to monitor a broiler’s under-wing temperature. However, it has the following demerits. Firstly, frequent replacement of the battery is required. Secondly, it is difficult to keep the sensor in a fixed position under the wing. Position shift of the sensor will cause incorrect under-wing temperature. Worse, falling off of the sensor could lead to temperature data loss.

In recent years, thermal imaging technology has been successfully applied to obtain superficial temperature for cows [9], cattle [10], pigs [11], and so on. Based on the obtained superficial temperature, animal health was evaluated [12,13]. In the field of poultry breeding, thermal imaging has also been employed to obtain the surface temperature for broilers. For example, Giloh, Shinder, and Yahav [1] extracted broiler surface temperature from a thermal image. They found that broiler surface temperature is correlated to its body temperature. Liu, et al. [14] established a regression model between surface and core body temperatures for an individual broiler. However, broiler surface temperature in the existing research was extracted manually by using the specific software provided by the thermal imager manufacturer [15]. This is time and labor consuming, especially when the number of images is large and the ROI (region of interesting) areas are small.

The broiler’s head has few feathers, it is much easier to obtain the skin temperature in head than other parts of an individual broiler by using a top-view thermal camera. Inspired by the study done by Giloh, et al. [1] and Liu, et al. [14], an automatic head surface temperature extraction algorithm for top-view thermal image with individual broiler was developed in this study. At the same time, a method for head temperature time series smoothing was proposed, based on which, febrile broiler can be identified. This research lays a foundation for the development of an automatic system for febrile broiler identification.

## 2. Materials and Methods

### 2.1. Image System

**Figure 1:**
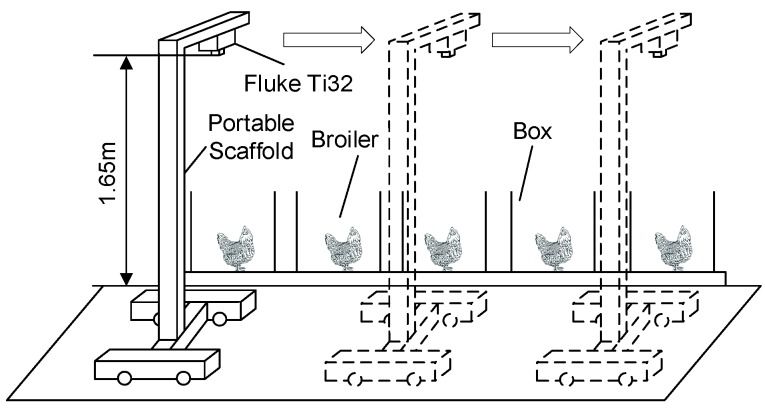
Image system.

The image system consisted of a portable scaffold and an infrared thermal camera (Fluke TI32, Avery, WA, USA), as shown in Figure 1.

The image resolution, thermal sensitivity, field of view (FoV) of the thermal camera was 320 × 240, ≤0.045 °C at 30 °C, and 23° × 17°, respectively. Five paper boxes were placed on the ground, each one with a broiler inside and with no cover. The camera was fixed on the portable scaffold at a height of 1.65 m above the ground. FoV of the camera at this height allows for coverage of the whole boiler in each paper box. According to the survey conducted by McManus, et al. [16] in 2016, emissivity value normally ranged from 0.98 to 0.93 for animal temperature monitoring. Therefore, the emission rate in this study was set to 0.95. The portable scaffold was pushed manually and slid from one box to another. Once the portable scaffold stopped beside a box, thermal imager was triggered manually to capture thermal images for the broiler inside the box. The thermal images were stored in the SD card of the thermal imager in IS2 format. Each IS2 file contained the radiometric data, infrared image, IR-Fusion mode information, and so on, of the object in the FoV of the imager. At the end of thermal image acquisition, all the obtained IS2 files were transferred to a PC for further processing.

### 2.2. Broilers and Thermal Images Acquisition

20 QingJiaoMa broilers, with the age of around 45 days (45.3 days ± 1.23 days) and a body weight of around 1.5 kg (1.54 kg ± 0.12 kg) were randomly selected from Lihua Animal Husbandry Co. LTD, city of Changzhou, Jiangsu Province, China. All the selected broilers were confirmed to be healthy by a veterinarian. Thermal images of the broilers were acquired in an experimental animal house of Lihua Animal Husbandry Co. LTD from 19 September 2018 to 24 September 2018. Ambient temperature inside the experimental animal house was set to 27–30 °C by using an air conditioner. The environmental relative humidity was 55–65%. Three fluorescent lamps equipped on the ceiling were used to provide stable illumination conditions inside the house. There was no thermal heat from solar or other light sources during the whole course of the thermal images acquisition.

15 broilers were randomly chosen and served as the experimental group. Thermal images of the broilers in this group were captured in 3 batches (5 broilers in each batch). The remaining 5 broilers served as the control group in this study and were photographed in one batch. Thermal image acquisition began at 8:30 a.m. and ended at 11:00 p.m. each day. For each broiler in the experimental group, 0.1 mL of the pasteurella inoculum was injected into its breast at 12:00 a.m. For broilers in the control group, no treatment was given. A wireless wearable sensor based under-wing temperature monitoring system (CH-T2, Chero Technology Co. ltd, Zhejiang, China) was utilized to collect the under-wing temperature for each broiler. The temperature sensor of this system was positioned under each broiler’s wing the night before the next day’s thermal images acquisition. After the sensor installation was completed, each broiler was placed in one of the 5 boxes overnight to help it adapt to the experimental environment and the sensor under its wing. The under-wing temperature of each broiler was collected by the temperature sensor and sent to the application installed in a smart phone at a fixed time interval.

The inherent error between the actual and the monitoring temperature obtained by the thermal camera used in this study was ±2 °C. Averaging operation was employed to reduce the impact brought on by the inherent error. Therefore, five thermal images were captured for each broiler every 30 min. Each broiler of the control and experimental groups was photographed for 30 cycles and 15–21 cycles, respectively, where the quantity of the thermal image acquisition cycles of the broilers in experimental group depended on its dead time. At the end of thermal image acquisition, an image database with 2185 thermal images was procured.

### 2.3. Head Surface Temperature Extraction

An algorithm, named *HSTE* (Head Surface Temperature Extraction), was developed to extract head surface temperature from a top-view thermal image with individual broilers. *HSTE* consisted of 3 steps: thermal image pre-processing, head region locating, and representative head surface temperature extraction.

#### 2.3.1. Thermal Image Pre-Processing

**Figure 2:**
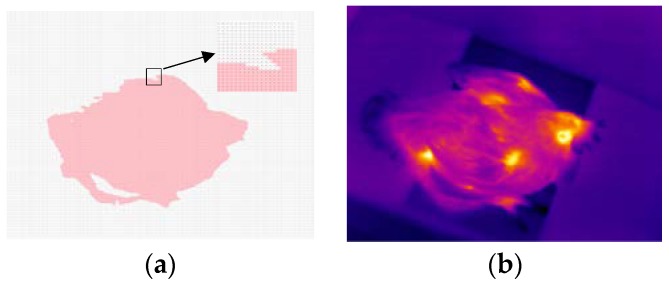
Temperature matrix and thermal image of an IS2 file: (**a**) Temperature matrix; (**b**) The corresponding thermal image.

Each thermal image obtained in Section 2.2 was converted to a 240 × 320 matrix. The matrix contained the temperature value at each pixel. Taking a thermal image selected randomly from the image database as an example, its temperature matrix and thermal image are shown in Figure 2a,b, respectively.

**Figure 3:**
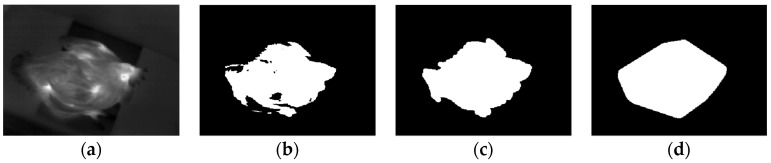
Image pre-processing: (**a**) Grayscale image; (**b**) Binary image; (**c**) Image after morphological processing; (**d**) Convex hull image.

Temperature matrix shown in Figure 2a was transformed into a grayscale image, as shown in Figure 3a, by using Formula (1):

(1) $$Img\_gray\left[r,c\right]=\frac{Img\_m\left[r,c\right]-min\left(Img\_m\right)}{max\left(Img\_m\right)-\;min\left(Img\_m\right)}\;r\in \left[1,240\right],\;c\in \left[1,320\right].$$

where *Img_gray*[*r*, *c*] was the intensity value of the pixel at the coordinate (*r*, *c*) in the grayscale image. *Img_m*[*r*, *c*] was the value at row *r* and column *c* of the temperature matrix, *max*(*Img_m*) and min(*Img_m*) were the maximum and minimum value of the temperature matrix, respectively.

The obtained grayscale image was converted to a binary one by using Otsu’s method [17], as shown in Figure 3b. Then, morphological processing [18] was carried out to remove holes and spindly parts in the binary image. The morphological processing result of Figure 3b is shown in Figure 3c. Finally, the convex hull image was obtained by using the algorithm proposed by Barber, et al. [19] in 1996, as shown in Figure 3d.

#### 2.3.2. Head Region Locating

The contour of a broiler can be approximately considered an ellipse, where head part is definitely located nearby one of the two endpoints of the major axis of the ellipse. At the same time, there are few feathers in a broiler’s head, leading to a relatively high temperature region in the head part of a thermal image. Therefore, head region was located in a thermal image based on the features of surface temperature distribution and a rough elliptical body shape according to the following 3 sub-steps.

**Figure 4:**
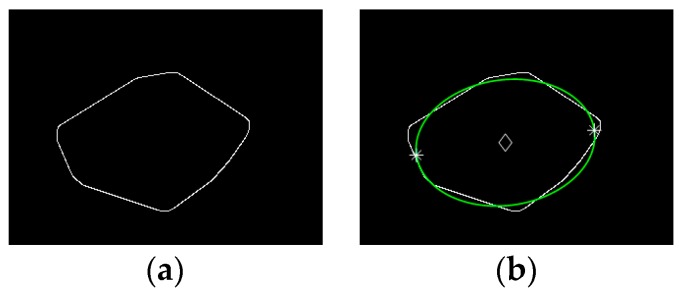
Ellipse fitting for individual broiler body contour: (**a**) The contour of the convex hull image shown in Figure 3d; (**b**) The fitted ellipse.

Sub-step 1; coordinates of the major axis endpoints extraction for the fitted ellipse. An ellipse was fitted for the convex hull image contour of an individual broiler by using direct least square method [20,21]. The fitted ellipse of the contour in Figure 4a is shown in Figure 4b, where the contour in Figure 4a was extracted from the convex hull image in Figure 3d by using the method proposed by Canny [22] in 1986. Once the fitted ellipse was obtained, the following parameters of the ellipse were extracted. The coordinate of the center point, which was denoted by $\left(x_{0}^{\prime },y_{0}^{\prime }\right)$ is shown in Figure 4b with a diamond point. Length of the major and minor axis were denoted by *a* and *b*, respectively. The inclined angle of the ellipse’s major axis was denoted by α. Based on the aforementioned parameters, coordinates of the two endpoints of the major axis were calculated by Equations (2)–(5):

(2) $$x_{1}=\frac{a}{2}cos\alpha +x_{0}^{\prime }$$

(3) $$y_{1}=\;\frac{b}{2}sin\alpha +y_{0}^{\prime }$$

(4) $$x_{2}=-\frac{a}{2}cos\alpha +x_{0}^{\prime }$$

(5) $$y_{2}=-\frac{b}{2}sin\alpha +y_{0}^{\prime }$$

where *x*_1_ and *x*_2_, *y*_1_, and *y*_2_ were the horizontal and vertical coordinates of the two endpoints, respectively. The extracted endpoints are shown in Figure 4b with two star points.

**Figure 5:**
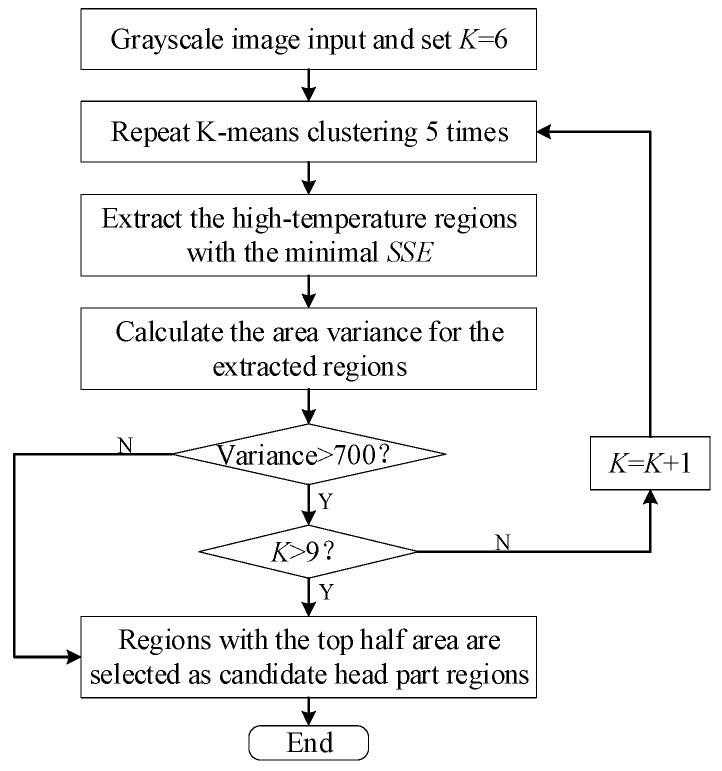
Flowchart of the candidate head regions extraction.

Sub-step 2, candidate head regions extraction. Figure 2a indicates that there are several high intensity regions in a broiler’s grayscale image. The higher the intensity of a region, the higher the temperature in this region. The head, which has no feather or just sparse feathers, is definitely among these high intensity regions. Adaptive K-means clustering method was carried out to locate these high intensity regions. Then, high intensity regions with the top half area were extracted as the candidate head regions. The main operation of candidate head regions extraction is indicated in the flowchart shown in Figure 5.

Parameter *K* of the adaptive K-means clustering [23] was initialized to be 6. That is, 6 pixels in the grayscale image shown in Figure 3a were randomly selected as the centroids. The Manhattan distance [24], calculated by Equation (6):

(6) $$d_{pd}=\left|x_{p}-c_{q}\right|\;p\in \left[1,76800\right],\;q\in \left[1,K\right],\;$$

was applied to measure the intensity distance from the rest of the pixels to the 6 centroids. *x_p_* was the intensity of the pixel in row (*p* mod 240), column (*p*/240 + 1) of the grayscale image. “/” and “mod” were modulus and remainder operators on integer values, respectively. *c_q_* was the intensity of the *q*th centroid. Operator |*w*| referred to the operation of extracting the absolute value of *w*.

A maximum of 4 iterations of K-means clustering were carried out to obtain the high intensity regions, where each iteration had 5 clustering operations. That is, 5 sets of temporary high intensity regions were obtained for each iteration. The sum of square error, denoted by *SSE* and calculated by Equation (7):

(7) $$SSE=\overset{M}{\underset{p=1}{\sum }}\sum_{q=1}^{K}d_{pq}{}^{2}$$

was utilized to choose the result high temperature regions for each iteration. Where, *M* was set to be 240 × 320 and *K* was adjustable.

**Figure 6:**
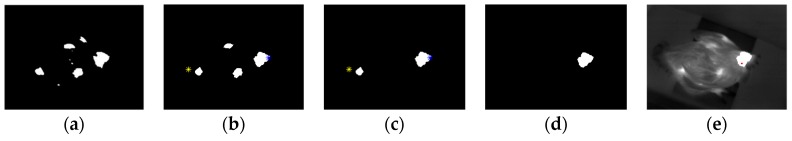
Head region locating: (**a**) The final high temperature regions extracted from the image in Figure 2a; (**b**) The candidate head regions; (**c**) The alternative head regions; (**d**) The extracted head region; (**e**) Relationship between the extracted head region and the gray-scale thermal image.

Among the 5 sets of temporary high intensity regions, the one with the minimal *SSE* was selected as the result high intensity regions for the current iteration. The variance of the areas of the resulting high intensity regions was calculated. If the variance was smaller than 700, the resulting high intensity regions were chosen as the final high intensity regions. Otherwise, if *K* was smaller than 9, a new iteration of clustering was implemented with *K* increased by 1. Clustering terminated when either the variance was smaller than 700 or *K* was greater than 9. The final high intensity regions of the grayscale image shown in Figure 3a after the adaptive K-means clustering operation is shown in Figure 6a.

The area of each final high intensity region was calculated. Regions with the top half areas were extracted as the candidate head regions. The candidate head regions of Figure 6a are shown in Figure 6b, where the star points are the endpoints of the major axis of the fitted ellipse. The two endpoints were denoted by *EP1* and *EP2*, respectively.

Sub-step 3, head region locating. The broiler’s head must be close to one of the endpoints of the major axis of the fitted ellipse of its top-view body contour. Therefore, two of the candidate head regions, each of which was closest to one of the endpoints of major axis of the fitted ellipse, were selected as the alternative head regions. The alternative head regions selected from the candidate head regions in Figure 6b are shown in Figure 6c. Head region was chosen from the two alternative head regions, denoted by *L1* and *L2*, respectively, according to the following 3 cases:

**Case 1**: *EP1* and *EP2* located inside *L1* and *L2*, one endpoint in one region. Then, the alternative head region with higher maximum temperature was extracted as the head region.

**Case 2**: Only one endpoint located inside *L1* or *L2*. Then, the alternative head region, in which the endpoint was located, was extracted as the head region.

**Case 3**: No endpoint located inside *L1* or *L2*. Then, the shorter distance of each endpoint to the center of *L1* and *L2*, denoted by *D_min1* and *D_min2*, respectively, were calculated and obtained. The alternative head region with a distance of *min*{*D_min1*, *D_min2*} s extracted as the head region. Where, *min*{*D_min1*, *D_min2*} was the smaller value of *D_min1* and *D_min2*.

**Figure 7:**
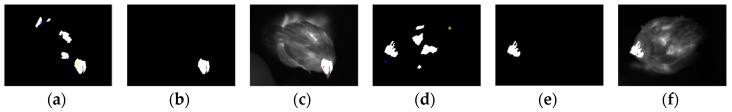
Examples of the head region locating: (**a**–**c**) Head region locating by using case 1; (**d**–**f**) Head region locating by using case 3.

Taking the alternative head regions in Figure 6c as an example, head region was located by using Case 2, as shown in Figure 6d. The relationship between the extracted head region and the original gray-scale thermal image is shown in Figure 6e. Examples of the head region located by using case 1 and case 3 are shown in Figure 7b,e, respectively.

Where the corresponding candidate head regions are shown in Figure 7a,d, respectively. The relationship between the extracted head region and the gray-scale thermal image is shown in Figure 7c,f. Operations described in Section 2.3.1 and Section 2.3.2 were applied to all the 2185 thermal images in the database to locate the broiler’s head region. As a result, the head region of the 2027 thermal images were located correctly.

Once the head region was located, the maximum temperature in the region was extracted as the head surface temperature, which was denoted by *HT_i_*_,*j*,*k*_ (*i* ∈ [1, 20]). Where *i* was the number of the broiler and *j* was the number of the thermal image capture interval. For a broiler in the experimental group, the maximum value of *j* depended on its dead time. For broilers in the control group, the maximum value of *j* was 30. *k* was the number of the thermal image in each interval. It had a maximum value of 5.

According to the technical manual of the thermal imager used in this study, the maximum error between the temperatures recorded by the thermal imager and animal’s actual superficial temperature is ±2 °C. In order to reduce the influence of this inherent error, representative head surface temperature (abbreviated as *RHT*) of the *i*th broiler in *j*th internal was calculated by Equation (8):

(8) $$RHT_{i}^{j}=(\sum_{k=1}^{N_{j}}HT_{i,j,k})\;/\;N_{j}\;i\in \left[1,20\right]$$

where *N_j_* is the quantity of the thermal images in the *j*th interval.

#### 2.3.3. Construction of RHT Time Series

**Figure 8:**
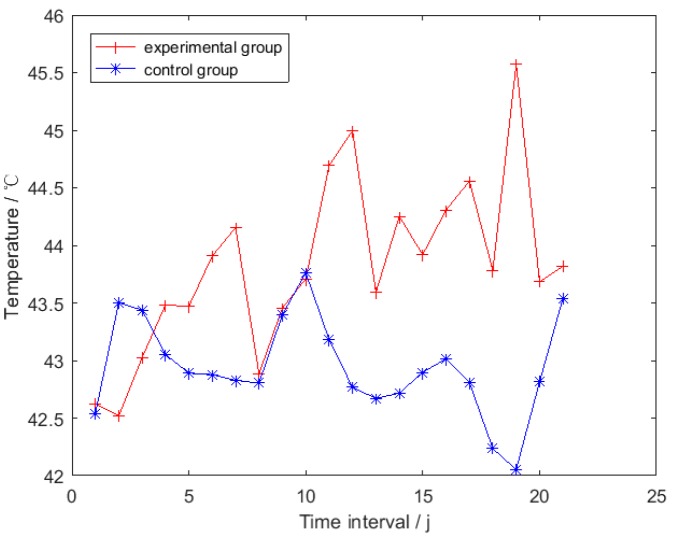
Representative head surface temperature (*RHT*) time series.

Equation (8) was applied to all the 2027 thermal images obtained in Section 2.3.2. A *RHT* time series, which was denoted by *TSRHT*, could be constructed for each broiler. Taking the *i*th broiler as an example, its *TSRHT* was obtained by using expression (9):

(9) $$TSRHT_{i}=\left\{RHT_{i}^{1},RHT_{i}^{2},\ldots ,RHT_{i}^{j},\ldots ,\;RHT_{i}^{NIH_{i}}\right\}\;i\in \left[1,20\right].$$

where *NIH_i_* is the quantity of the intervals in the thermal images acquisition step for the *i*th broiler. Two broilers, one in the experimental group and another in the control group, were randomly selected, the corresponding *TSRHT* of which are shown in Figure 8.

**Figure 9:**
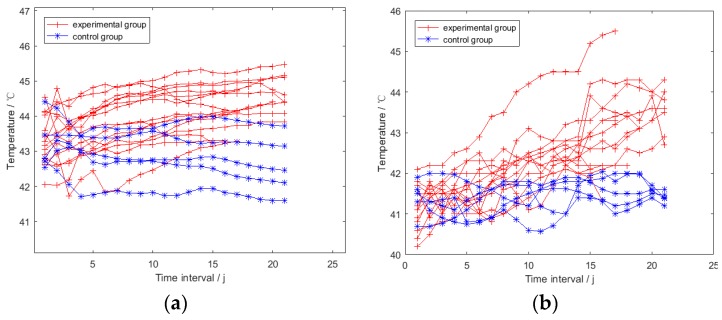
The smoothed representative head surface temperature time series (*TSRHT*) and under-wing temperature time series: (**a**) The smoothed *TSRHT* time series; (**b**) Under-wing temperature time series.

Data points in the *TSRHT* of the broiler in experimental and control groups are displayed with red cross and blue star points in Figure 8, respectively. It can be observed that sharp rising and drop exist in the whole *TSRHT*; even the average operation has been applied to reduce influence of the inherent error of the thermal imager. Therefore, the original *TSRHT* could not be utilized directly to identify febrile broiler. Smoothing operation was carried out to each *TSRHT* to obtain a gentle time series. Taking the *i*th broiler as an example, the *t*th data point in its smoothed *TSRHT_i_* (*i* ∈ [1, 20]) was obtained by calculating the mean of the first *t* (*t* ∈ [1, *NIH_i_*]) elements in *TSRHT_i_* by using Equation (10):

(10) $$m_{i}^{t}=\frac{\sum_{j=1}^{t}RHT_{i}^{j}}{t}\;t\in \left[1,NIH_{i}\right]\;,\;i\in \left[1,20\right].\;$$

where $m_{i}^{t}$ is the smoothed value of the *t*th data point in the smoothed *TSRHT_i_*. *NIH_i_* and $RHT_{i}^{j}$ are the quantity of the elements and the *j*th element in *TSRHT_i_*, respectively. The smoothed *TSRHT* of the broilers in the experimental and control groups are shown in Figure 9a with red cross and blue star points, respectively. The corresponding under-wing temperature time series is shown in Figure 9b.

The maximum time intervals of the broilers in the experimental group was 21; data points of broilers in control group from the 22nd to 30th time interval are not plotted in Figure 9. It can be observed in Figure 9a that the smoothed *TSRHT* of broilers in the experimental and control groups had an overall slight increase and flat trends, respectively. Meanwhile, under-wing temperature time series in Figure 9b indicates that almost all the broilers in the experimental groups were feverish (under-wing temperature greater than 42 °C) from the 13rd time interval on. All the broilers in the control group were not feverish (under-wing temperatures less than 42 °C). This is consistent with the result found by Meltzer in 1983 and by Donkoh in 1989. Both studies suggested that most healthy broilers had an ordinary body temperature of 40–42 °C [25,26]. Therefore, all the broilers in the experimental group in this study were infected successfully by the pasteurella inoculum. It can be inferred from Figure 9 that different trend features existed in the smoothed *TSRHT* of febrile and non-febrile broilers, which can be utilized to identify individual febrile broilers.

## 3. Results

### 3.1. Testing of HSTE

**Figure 10:**
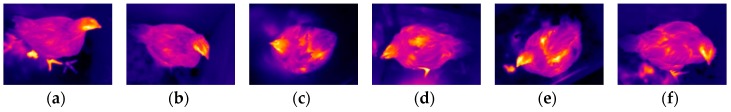
Example image of each category: (**a**–**f**) Example images of category (i)–(vi).

Operations described in Section 2.3.1 and Section 2.3.2 were applied to all the 2185 thermal images to locate a broiler’s head region. Thermal images were classified into the following six categories: (i) broiler body was well covered by feathers, and both feet and uncooled manure appeared; (ii) broiler body was well covered by feathers, and only feet or only uncooled manure appeared, or no feet and uncooled manure appeared; (iii) broiler body had sparse feathers, no feet, and uncooled manure appeared; (iv) broiler body had sparse feathers, and feet appeared; (v) broiler body had sparse feathers, an uncooled manure appeared; and (vi) broiler body had sparse feathers, and both feet and uncooled manure appeared. An example image for each category is shown in Figure 10.

Algorithm testing results indicated that the head region of the thermal images in category (a)–(f) could be correctly located with a ratio of 78.05%, 94.90%, 94.94%, 91.53%, 84.89%, and 73.68%, respectively, as shown in Table 1.

As a result, the average ratio of the correct locating of the head region was 92.77%. That is, the head region of 2027 thermal images were located correctly. These images were picked out for the *TSRHT* construction described in Section 2.3.3.

**Figure 11:**
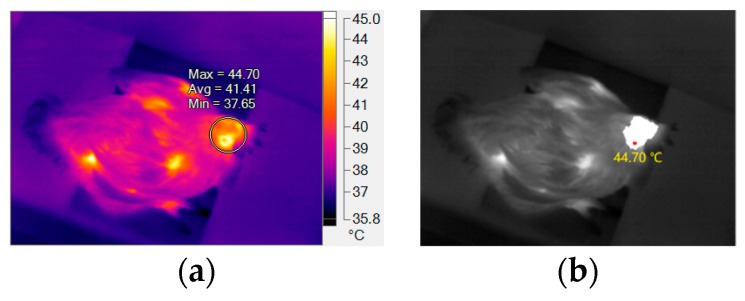
Head temperature extraction: (**a**) Head temperature extracted manually by using Smartview; (**b**) Head temperature extracted automatically by *HSTE*.

In order to evaluate the accuracy of the head temperature obtained automatically by extracting the maximum value in the head region, 100 thermal images were randomly selected from the 2027 images whose broiler head regions were located correctly. Head temperatures of the selected thermal images were obtained automatically and manually by using *HSTE* and Fluke Smartview 4.3, respectively. In the latter case, head region was selected by drawing a circle in the head for each selected thermal image. SmartView 4.3 provided the maximum, mean, and minimum temperatures of the selected region, as shown in Figure 11a.

**Figure 12:**
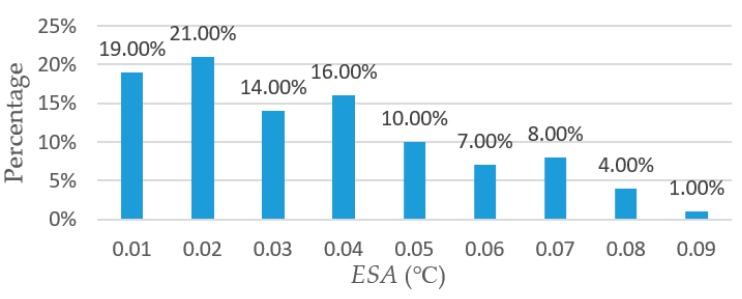
The errors between head temperatures extracted automatically by *HSTE* and by using Smartview.

Head temperature extracted automatically for the thermal image in Figure 11a is shown in Figure 11b. Using the maximum temperature in the selected region provided by the SmartView 4.3 as the gold standard, the accuracy of the head surface temperature extracted by *HSTE* was evaluated. For the thermal images whose broiler head part was correctly located, correlation coefficient between the maximum temperatures extracted by *HSTE* (38.86 °C ± 3.77 °C) and by Smartview (38.9 °C ± 3.76 °C) was 99.99%. The standard deviation of the maximum temperatures was close to 4 °C; this is because the thermal images were randomly selected from different broilers in different groups (including experimental and control groups). The absolute value of the error of the head surface temperature was extracted automatically and by SmartView 4.3 was denoted by *ESA*. For all the selected 100 thermal images, *ESA* is shown in Figure 12.

Figure 12 indicates that the maximum *ESA* of the head temperature, extracted automatically and manually, is less than 0.1 °C. Where 95% of the *ESA* are distributed in the range of 0.01–0.07 °C, it can be concluded that once the head region is located correctly, the head temperature can be extracted automatically with an acceptable accuracy.

### 3.2. Overall Trend Analysis for the Smoothed TSRHT

It was observed in Figure 9a that overall trends of slight increase and flat existed in the smoothed *TSRHT* of broilers in experimental and control groups, respectively. Slope of the straight line fitted by the first *t*′ (*t*′ ∈ [5, *NIH_i_*], i∈[1,20]) elements in a smoothed *TSRHT_i_*, which was denoted by $Slope\_TSRHT_{i}^{t^{\prime }}$, was used to describe these two different overall trend features. For the *i*th broiler, all of its $Slope\_TSRHT_{i}^{t^{\prime }}$ formed a slope time series. This series was denoted by $Slope\_TSRHT_{i}$ and expressed in expression (11).

(11) $$Slope\_TSRHT_{i}=\left\{Slope\_TSRHT_{i}^{5},\ldots ,Slope\_TSRHT_{i}^{t^{\prime }},\ldots ,\;Slope\_TSRHT_{i}^{NIH_{i}}\right\}\;t^{\prime }\in \left[5,\;NIH_{i}\right],\;i\in \left[1,20\right]$$

**Figure 13:**
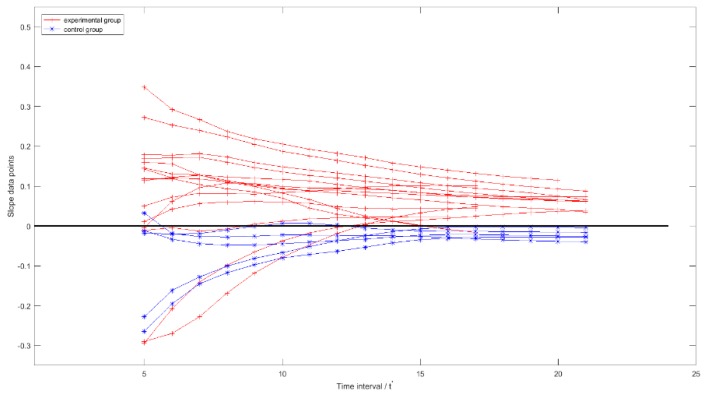
Slope series of each smoothed *TSRHT_i._*

As the maximum and minimum value of *NIH_i_* respectively was 15 and 21, quantity of the elements in each $Slope\_TSRHT_{i}$ was between 11 and 17. $Slope\_TSRHT_{i}$ of all the 20 broilers are plotted in Figure 13.

Where data points of the broilers in the experimental and control groups are displayed with red cross and blue star points, respectively, the black line is a boundary, which separates slope data points into positive and non-positive groups. For each given *t*′, the percentage of all the $Slope\_TSRHT_{i}^{t^{\prime }}$(*i* ∈ [1,20]) in the experimental and control groups that respectively had positive and non-positive values were listed in Table 2.

Preliminary statistical results in Table 2 indicated that from the 13^th^ time interval on, almost all the $Slope\_TSRHT_{i}^{t^{\prime }}$ of the smoothed *TSRHT* at each *t*′ in control and experimental groups had non-positive and positive values, respectively. It could be concluded that the positive or non-positive of the $Slope\_TSRHT_{i}^{t^{\prime }}$ can be used to identify whether a broiler is febrile at a given time interval.

## 4. Discussion

An algorithm was developed to extract head surface temperature from top-view thermal image with an individual broiler. Algorithm testing result indicated that head regions in 7.23% of the thermal images were wrongly located. This would bring about a wrong head temperature, which would in turn influence the trend feature of the smoothed *TSRHT*. Therefore, thermal images, whose head region were wrongly located, were picked out manually before *RHT* was calculated. This set a barrier against the automatic identification of the febrile broiler. Aiming at this insufficiency, the following two possible solutions are worthy of further study in the future.

The first possible solution is to introduce more image information for head region locating. RGB and thermal cameras can be integrated together to capture RGB and thermal images for a broiler at the same time and from the same angle. Head region can be located based on the fused RGB and thermal images. Where the color of the comb, the shape, and context characters of the boiler head, combined with the fitted ellipse and the surface temperature distribution of the broiler body, can be integrated to improve the ratio of the correct locating of head region. The second possible solution is to identify febrile broiler based on the average temperature time series of all the high temperature regions in a broiler’s body surface. High temperature regions can be located much easier than the head region. However, the relationship between the under-wing temperature and the feature of the average temperature time series of the high temperature regions is still unknown and deserving further study.

It is important to note that the ambient temperature and relative humidity were respectively 27-30 °C and 55–65% in this study. Both of the environmental parameters were stable during the whole process of the thermal images collection. A great deal of studies indicated that ambient temperature and relative humidity had impacts on the animal’s superficial temperature [11]. That is to say, head surface temperature should be considered a dependent variable on several independent variables, such as animal body temperature, ambient temperature, relative humidity, and so on. However, for a different ambient temperature and relative humidity range, as long as the environmental parameters are stable, the same impact would be brought to all the data points in a smoothed *TSRHT*. That is to say, a different ambient temperature and relative humidity range would not influence the overall trend of the smoothed *TSRHT*. Therefore, head surface temperature extracted by *HSTE* should be suitable for febrile broiler identification in different ambient temperatures, as long as the variation range of the environment parameters are small.

Furthermore, it can be observed from Figure 13 that some non-positive data points appeared in the smoothed *TSRHT* of the broilers in the experimental group in the first several time intervals. If the positive or non-positive of $Slope\_TSRHT_{i}^{t^{\prime }}$ is used to identify whether a broiler is febrile, a method to determine the start time of the febrile broiler identification should be developed in further study. Finally, different trend features were found in the smoothed *TSRHT* of febrile and non-febrile broilers. It can be inferred that a different proportion of febrile broilers in a broiler flock could bring different features of the population mean superficial temperature time series. Combined with the adhesive animal image segmentation methods [21,27,28], *HSTE* could be employed to extract population mean head surface temperature for broiler flocks. Therefore, the algorithm developed in this research can be regarded as a first step of the development of an automatic warning system for febrile broiler flocks.

## 5. Conclusions

Ellipse fitting and adaptive K-means clustering were integrated to extract broiler head surface temperature from top-view thermal image with individual broilers in this study. Algorithm testing results indicated that 92.77% of the thermal images could be processed correctly, and the maximum error of the extracted head temperatures was less than 0.1 °C. For the thermal images whose broiler head part was correctly located, correlation coefficient between the maximum temperatures extracted by the developed algorithm and by Smartview was 99.99%. Different overall trend features were observed in the smoothed representative head surface temperature time series (*TSRHT*) of febrile and non-febrile broilers. A slope time series was constructed based on the slopes of the straight lines fitted by the first several data points in each smoothed *TSRHT*. Preliminary statistical analyses were carried out for the signs (positive and non-positive) feature of these slope time series, and the result indicated that the signs feature of the data points in a slope time series can be used to identify whether a broiler is febrile or not. The presented method lays a foundation for the development of an automatic system for febrile broiler identification.

## Figures and Tables

**Table 1:** Ratio of correct locating of head region for images in different categories.

Category	(i)	(ii)	(iii)	(iv)	(v)	(vi)
Number of images	41	785	849	295	139	76
Number of correct locating	32	745	806	270	118	56
Ratio of correct locating	78.05%	94.90%	94.94%	91.53%	84.89%	73.68%

**Table 2:** Percentage of positive and non-positive values for different *t*′.

*t*′	5	6	7	8	9	10	11	12	13	14	15	16	17	18	19	20	21
***E***(%)	73	80	80	80	86.7	86.7	86.7	86.7	100	100	100	92.9	92.3	100	100	100	100
***C***(%)	80	100	100	100	100	80	80	80	100	100	100	100	100	100	100	100	100

***E***: experimental group; ***C***: control group.
